# Integrating RNA-Seq With GWAS Reveals a Novel SNP in Immune-Related *HLA-DQB1* Gene Associated With Occupational Pulmonary Fibrosis Risk: A Multi-Stage Study

**DOI:** 10.3389/fimmu.2021.796932

**Published:** 2022-01-17

**Authors:** Yan Zhou, Yingyi Zhang, Rui Zhao, Zhounan Cheng, Minzhu Tang, Anni Qiu, Yang Dong, Yihua Lu, Yulong Lian, Xun Zhuang, Tian Tian, Wei Wang, Minjie Chu

**Affiliations:** ^1^ Department of Epidemiology, School of Public Health, Nantong University, Nantong, China; ^2^ Department of Occupational Disease, The Eighth People’s Hospital of Wuxi, Wuxi, China; ^3^ Department of Respiratory, The Eighth People’s Hospital of Wuxi, Wuxi, China; ^4^ Department of Occupational Health, Center for Disease Control and Prevention of Wuxi, Wuxi, China

**Keywords:** silicosis, mRNA, *HLA-DQB1*, rs9273410, susceptibility

## Abstract

**Objective:**

To evaluate the association between single-nucleotide polymorphisms (SNPs) in RNA-seq identified mRNAs and silicosis susceptibility.

**Methods:**

A comprehensive RNA-seq was performed to screen for differently expressed mRNAs in the peripheral blood lymphocytes of eight subjects exposed to silica dust (four silicosis cases and four healthy controls). Following this, the SNPs located on the shortlisted mRNAs, which may affect silicosis susceptibility, were screened through silicosis-related genome-wide association studies (GWAS) (155 silicosis cases and 141 healthy controls), whereas functional expression quantitative trait locus (eQTL)-SNPs were identified using the GTEx database. Finally, the association between functional eQTL-SNPs and silicosis susceptibility (194 silicosis cases and 235 healthy controls) was validated.

**Results:**

A total of 70 differentially expressed mRNAs (fold change > 2 or fold change < 0.5, *P* < 0.05) was obtained using RNA-seq. Furthermore, 476 SNPs located on the shortlisted mRNAs, which may affect silicosis susceptibility (*P* < 0.05) were obtained using GWAS, whereas subsequent six functional eQTL-SNPs were identified. The mutant A allele of rs9273410 in *HLA-DQB1* indicated a potential increase in silicosis susceptibility in the validation stage (additive model: odds ratio (OR)= 1.31, 95% confidence interval (CI) = 0.99–1.74, *P* = 0.061), whereas the combination of GWAS and the validation results indicated that the mutant A allele of rs9273410 was associated with increased silicosis susceptibility (additive model: OR = 1.35, 95% CI =1.09–1.68, *P* = 0.006).

**Conclusion:**

The mutant A allele of rs9273410 was associated with increased silicosis susceptibility by modulating the expression of *HLA-DQB1*.

## Introduction

Silicosis is a chronic progressive fibrotic lung disease, which may lead to respiratory failure or death (1, 2). The Global Silicosis Elimination Plan has established the goal of reducing the impact of silicosis by 2030; however, silicosis remains a vital issue threatening the health of individuals in various occupations in both developing and developed countries. For instance, China records the largest incidence of silicosis in the world, with more than 873,000 silicosis cases being recorded by the end of 2018 (3). According to the systematic analysis of the Global Burden of Disease Study in 2016, East Asia ranks first in the world for the highest silicosis fatality rate followed by Western Europe (4). In addition, some Australian coal mining communities are facing a severe epidemic of accelerated silicosis due to artificial stone exposure (5). Therefore, silicosis remains a major global health concern (6).

Occupational exposure to siliceous dust is widely known to be the main cause of silicosis (7). Currently, no specific therapy exists for silicosis, apart from avoiding contact with environmental silica (8). Despite the escape from the harmful workplace, persistent lung function damage and disease progression are unavoidable owing to the difficulty in removing siliceous particles from the body (9). Another cause for concern is that individuals exposed to the same working environment might present different health conditions: some people suffer from silicosis after working in an exposed environment for several years, whereas others remain healthy (10). This indicates the possibility of potentially non-modifiable risk factors related to silicosis, such as genetic variants (mainly single-nucleotide polymorphisms, SNPs) (11).

Previous genome wide association studies (GWASs) have identified numerous pulmonary fibrosis-related SNPs, which were mainly idiopathic pulmonary fibrosis (IPF)-susceptible SNP-harbouring genes such as *MUC5B*, *AKAP13*, *DSP*, *FAM13A*, *DEPTOR*, *KIF15*, and *MAD1L1* (12–15). In addition, a three-stage GWAS had identified and replicated a genome-wide significant (*P* < 5.0 × 10^-8^) signal (named as variant rs73329476) that is associated with coal workers’ pneumoconiosis susceptibility and altered *CPM* expression (10). GWASs have proven to be successful in the discovery of SNPs associated with complex diseases; however, most SNPs identified using GWAS are in the gene desert regions, and their biological functions remain unclear. Moreover, developing post-GWAS methods for characterising the function of complex disease-associated SNPs is required. A recent report proposed that the integrative analysis of RNA sequencing (RNA-seq) data and GWAS mapping may be promising for illustrating the functional genetic architecture of underlying complex diseases (16), and the evidence that differentially expressed genes identified using RNA-seq were more likely to overlap with GWAS obtained loci have highlighted this strategy (17).

Recently, RNA-seq has become an indispensable and a powerful tool for transcriptome-wide analysis of differential gene expression, which quickly and comprehensively acquires approximately all transcriptional sequence information of a species at a certain stage (18). Studies suggest that RNA-seq can be employed to analyse the synergistic or antagonistic effects of various mRNAs associated with fibrotic lung diseases (19). A previous study identified 873 differentially expressed genes using RNA-seq from eight IPF lung samples and seven healthy controls (20). Another study using 3’ messenger RNA sequencing (mRNA-seq) and pathway enrichment analysis revealed the upregulation of pathways related to immune response and inflammatory signaling in patients with IPF compared with controls (19). A recent study on silicosis identified 600 upregulated genes and 537 downregulated genes in the silica inhalation-induced silicosis rat model group using RNA-seq and bioinformatic analyses (21). However, in silicosis human samples, RNA-seq was rarely used for the determination of specific differentially expressed genes.

In this study, we addressed the integration of RNA-seq data and GWAS mapping to effectively identify the potentially functional SNPs associated with the susceptibility of silicosis. First, RNA-Seq technology was used to identify mRNAs with distinct expression patterns among four pairs of silicosis cases and silica-exposed healthy controls. Second, candidate SNPs located on the RNA-seq-identified genes were selected, and the association between those SNPs and silicosis risk was evaluated using GWAS (155 silicosis cases and 141 healthy controls), while the potential functional SNPs with the expression quantitative trait locus (eQTL) regulation function were selected. Third, the association between the selected eQTL-SNPs and silicosis risk was validated using an additional case–control study (194 silicosis cases and 235 healthy controls).

## Methods and Materials

### Study Design

**Figure 1:**
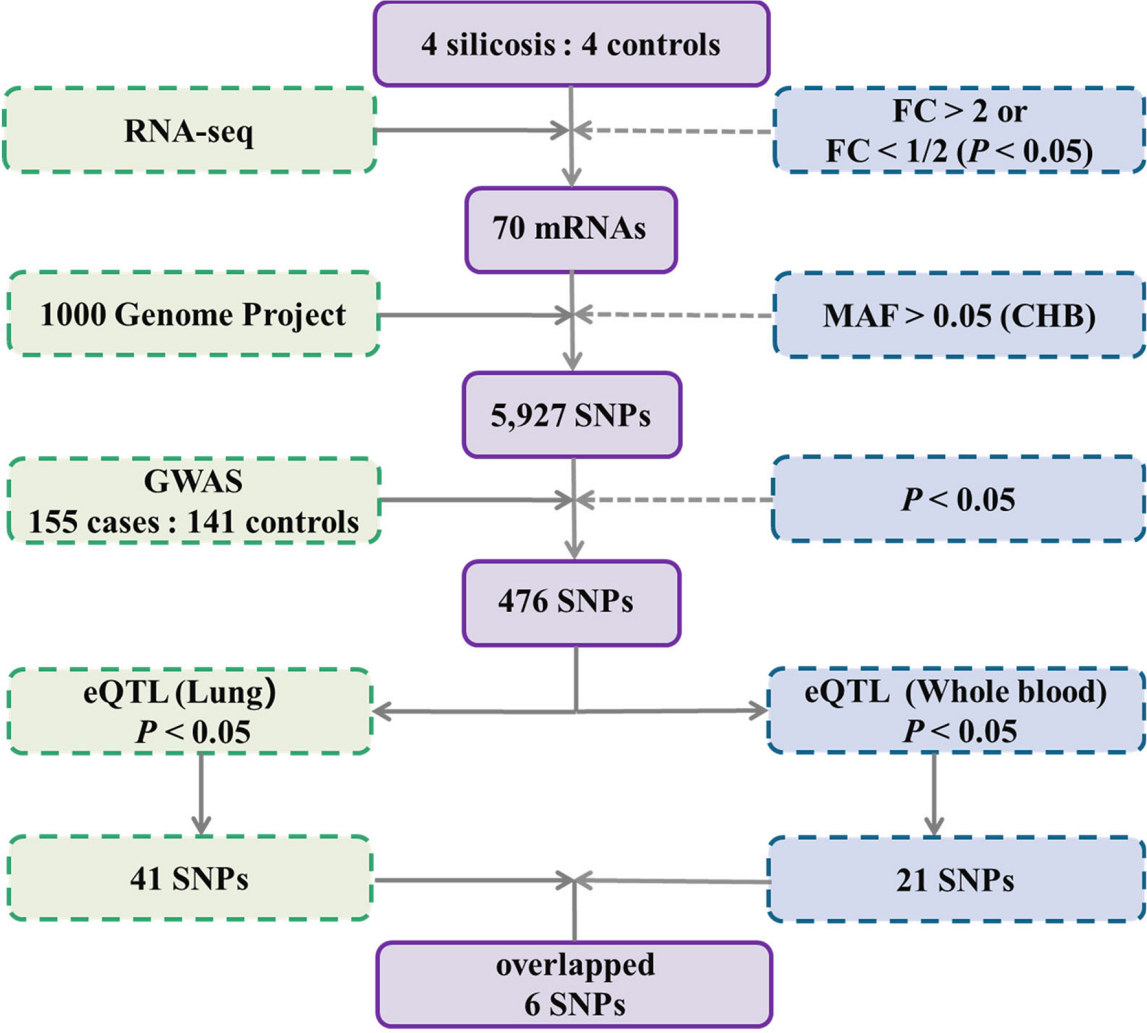
Schematic representation of the study design. RNA-seq, RNA sequencing; FC, fold change; MAF, minor allele frequency; eQTL, expression quantitative trait locus.

In the initial RNA-seq stage, differentially expressed mRNAs in the peripheral blood lymphocytes (PBLs) from four silicosis cases and four healthy controls with similar years of silica dust exposure and age were selected. All SNPs located on RNA-seq identified differentially expressed mRNAs with minor allele frequency (MAF) > 0.05 in the Han Chinese population were obtained using the 1000 Genome Project database. The silicosis-related GWAS (155 silicosis cases and 141 healthy controls with similar years of silica dust exposure) was used to screen for candidate positive SNPs. eQTL analysis in both the lung and whole blood was used to further screen for candidate functional eQTL-SNPs using the GTEx database. A linkage disequilibrium (LD) analysis (*r^2^* < 0.8) was performed among these SNPs using the Ensemble database. A validation study was designed (194 silicosis cases and 235 healthy controls with similar years of silica dust exposure) to validate the association between functional eQTL-SNPs and silicosis susceptibility (**Figure 1**).

### Study Population

To obtain the differently expressed silicosis-related mRNAs, a 1:1 individual matched case–control study was performed based on years of silica dust exposure and age. In principle, a case was generally matched to an exposed control with the most similar years of silica dust exposure (± 5) and age (± 5). Four patients with silicosis as the case group and four matched healthy individuals as the control group was selected in September 2017 from the Wuxi Institute of Occupational Diseases in Jiangsu Province.

The cases were diagnosed as stage I, stage II, or stage III silicosis according to the size, profusion, and distribution range of opacities on chest X-ray by three national certified occupational physicians based on the China National Diagnostic Criteria for Pneumoconiosis (GBZ 70-2015). The years of silica dust exposure for each individual were recorded in historical occupational health records.

The case group included in the GWAS screening stage was recruited in 2012–2016 from the Occupational Disease Institute of Wuxi. The control group of 141 healthy individuals was randomly selected from a pool of >2000 occupational dust exposed individuals who participated in the routine health surveillance of 2017 at Wuxi and were matched to the case group on the years of silica dust exposure.

In the improved Multiligase Detection Reaction (iMLDR) validation stage, 194 silicosis cases were recruited in 2017 from the Datun Mining Business Group Co. Ltd. of Xuzhou; 235 healthy controls were randomly selected from a pool of >1000 individuals with occupational silica dust exposure who participated in the routine health surveillance of 2017 in Xuzhou. These controls were matched to the cases based on the years of silica dust exposure.

Each participant filled out a structured questionnaire that included all demographic characteristics required for the study, with the help of administrative staff who are well-trained in conducting face-to-face interviews. All participants provided written informed consent, and the study protocol was approved by the Ethics Committee of Nantong University (Approval No. 2020-002).

### RNA-Seq Screening

From the PBLs of four patients with silicosis and four healthy controls, the total RNA was extracted using TRIzol (Invitrogen, Carlsbad, CA, USA) and a mRNeasy mini kit (Qiagen, Hilden, Germany), according to the manufacturer’s instructions. The RNA samples were outsourced to Gminix Biotechnology Co., Ltd. (Shanghai, China) for RNA-seq using the Illumina HiSeq 2500 sequencing platform that has an average of 15 G reads. Among the identified mRNAs, the differently expressed mRNAs between the four patients with silicosis and four healthy controls were selected with *P* < 0.05 and fold change (FC) > 2 (cases/controls: >2-fold up-regulated or <0.5-fold down-regulated).

### Genotyping Platform of the Two-Stage Case-Control Study

**Figure 2:**
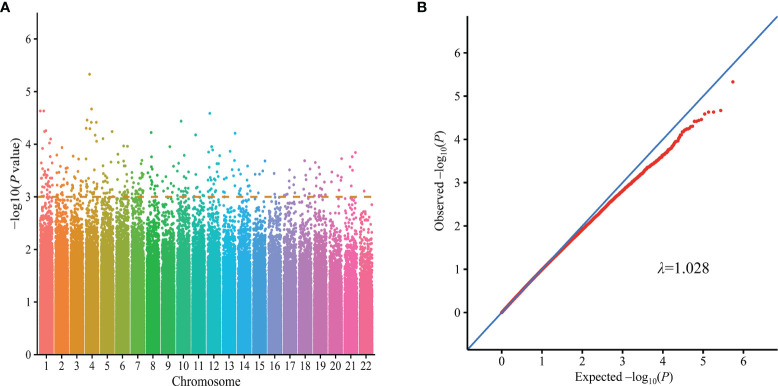
GWAS results on silicosis in Han Chinese population (155 cases and 141 controls). Manhattan plots represent -log10 (p-value) for SNPs distributed across all chromosomes **(A)**, and quantile-quantile (Q-Q) plot described the distribution of the observed (y-axis) and expected (x-axis) p-values of each SNPs **(B)**. Q-Q, quantile-quantile; GWAS, Genome-wide association study.

Genomic DNA was extracted from peripheral blood samples using a DNA extraction kit (Qiagen, Valencia, CA). A high throughput genotyping chip designed for the Asian population (Illumina Asian screening array chip) comprising 746,113 SNPs was used for genome-wide genotyping. After quality control, 155 cases and 141 controls with 544,414 autosomal SNPs were retained for subsequent analyses. And *P*-values from GWAS are described in Manhattan and Quantile-quantile plots (**Figures 2A, B**). Genotyping of the validation stage was performed using the Genesky proprietary iMLDR multiplex SNP genotyping system, which employs a multiplex PCR-ligase detection reaction method. For each SNP, the alleles were distinguished by different fluorescent labels of allele-specific oligonucleotide probe pairs. And then, different SNPs were further distinguished according to different extended lengths at the 3’end.

### eQTL Analysis

Using the GTEx database (http://www.gtexportal.org/home/), eQTL analysis was performed for the lung and whole blood tissues separately. Based on the overlapping SNPs between the two tissues, candidate SNPs with eQTL functions in both the lung and whole blood tissues were obtained.

### Statistical Analyses

Differences in the distribution of demographic characteristics and selected variables between the case and control groups were calculated using the two-sided chi-square tests and Student’s t-tests. Logistic regression analysis was performed to determine the association between candidate SNPs and silicosis risk based on the adjusted odds ratios (ORs) and 95% confidence intervals (CIs), adjusting for sex, age, smoking status, and silica dust exposure years. Generally, for each SNP, we assigned those carrying no minor allele to the wild-type homozygote (code 0), carrying one minor allele to the heterozygote (code 1), and carrying two minor alleles to the variant homozygote (code 2), and then we assessed the association in different genetic models: heterozygote (co-dominant) model (1 versus 0), homozygote (co-dominant) model (2 versus 0), dominant model (1 + 2 versus 0), recessive model (2 versus 1 + 0). For additive model, testing is designed specifically to reveal associations that depend additively on the minor allele. That is, subjects carrying two minor alleles (as compared with those carrying no minor allele) are twice as likely to affect the outcome in a certain direction as subjects carrying one minor allele (as compared with those carrying no minor allele). All analyses were conducted using the following software: SPSS version 20.0, STATA version 12.0 and R version 3.6.2.

## Results

### Characteristics of the Study Subjects

The characteristics of the subjects in the control and case groups are shown in **Table 1**. Generally, the sex ratios and silica dust exposure years between the case and control groups were comparable (*P* > 0.05). The mean age of the case group was higher than that of the control group, and the case group had higher smoking rates than those of the control group (*P* < 0.05). In addition, smoking was more severe in the case group than in the control group (*P* < 0.05).

**Table 1:** Characteristics of the subjects enrolled in this study.

Variables	Screening (GWAS)			Validation (iMLDR)		
	Case	Control	*P*	Case	Control	*P*
	(N = 155)	(N = 141)		(N = 194)	(N = 235)	
Age, years (mean ± SD)	67.53 ± 8.24	60.25 ± 6.31	<0.001	68.73 ± 9.01	62.71 ± 11.74	<0.001
Exposure years (mean ± SD)	24.80 ± 7.00	23.72 ± 5.55	0.146	27.13 ± 8.16	23.58 ± 8.50	0.055
Sex, N (100%)			0.051			0.151
Male	138 (89.03)	114 (80.85)		189 (97.42)	222 (94.47)	
Female	17 (10.97)	27 (19.15)		5 (2.58)	13 (5.53)	
Smoking status, N (100%)			0.019			0.005
Ever	98 (63.23)	70 (49.65)		103 (53.09)	92 (39.15)	
Never	57 (36.77)	71 (50.35)		91 (46.91)	143 (60.85)	
Stage, N (100%)						
I	94 (60.65)			154 (79.38)		
II	51 (32.90)			28 (14.43)		
III	10 (6.45)			12 (6.19)		

### RNA-Seq Screening

**Figure 3:**
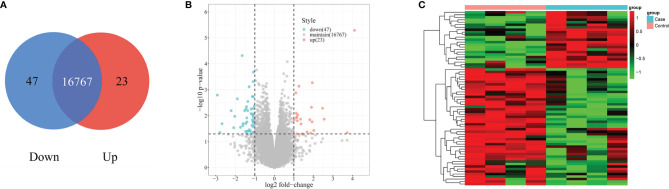
Differential expression of mRNAs in the case and control groups. **(A)** The Venn diagram shows 23 upregulated mRNAs and 47 downregulated mRNAs in the case group, and 16,767 mRNAs are maintained. **(B)** The differentially expressed mRNAs between the case and control groups are described using volcano plots. The red points represent the differentially expressed mRNAs on the statistical criteria of fold change >2-fold upregulated (log2 scaled) and *P* < 0.05 (−log10 scaled); the green points represent the differentially expressed mRNAs on the statistical criteria of fold change <1/2 downregulated (log2 scaled) and *P* < 0.05 (−log10 scaled). **(C)** Hierarchical clustering analysis of the 23 upregulated and 47 downregulated mRNAs.

RNA-seq detected 760 differently expressed mRNAs (*P* < 0.05) in the PBLs of four patients with silicosis and four healthy controls. A total of 70 differently expressed mRNAs with FC > 2 or FC < 0.5 were obtained from above 760 mRNAs. Among the 70 differentially expressed mRNAs, 23 had high expression and 47 had low expression (**Figure 3**).

### Selection of Promising SNPs in Candidate mRNAs

Using the 1000 Genome Project, 69,893 SNPs located on the 70 mRNAs obtained were screened out. A total of 5,927 SNPs were selected with a minor allele frequency (MAF) value > 0.05 in the Han Chinese population.

Subsequently, the silicosis related GWAS dataset was used to evaluate the association between the shortlisted 5,927 SNPs and silicosis susceptibility. The result revealed 476 SNPs to be significantly associated with silicosis risk (*P* < 0.05).

To evaluate the regulatory relationship between SNPs and corresponding mRNAs, further eQTL screening with the SNPs associated with silicosis risk was carried out. Among the 476 positive SNPs, 41 and 21 SNPs with different gene expressions were found in the lung tissue and whole blood tissues, respectively. Six overlapping SNPs in the lung and whole blood were eventually selected. LD analysis of the six SNPs showed no significant LD, and these SNPs were selected for use in the validation stage.

**Figure 4:**
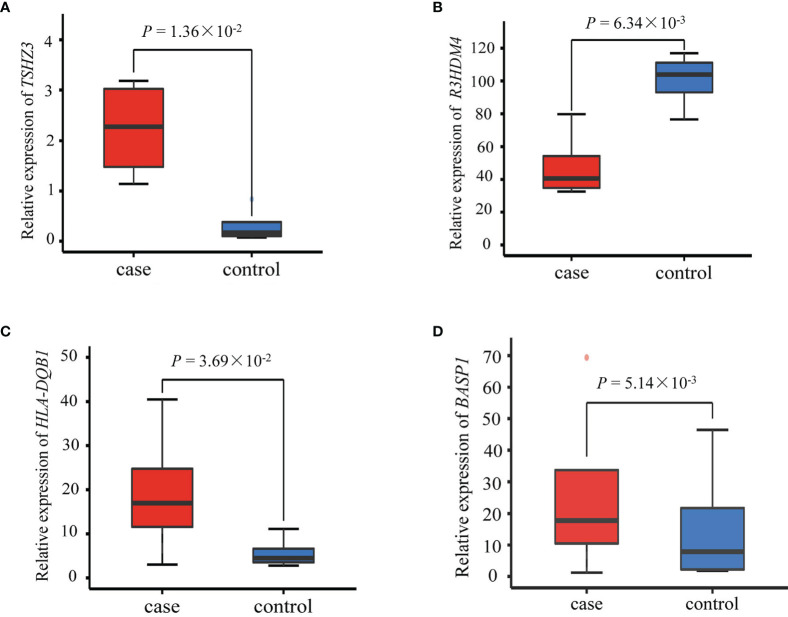
Differential expression of **(A)** *TSHZ3*, **(B)** *R3HDM4*, **(C)** *HLA-DQB1* and **(D)** *BASP1* between the four patients with silicosis and four healthy controls. *P*-value was calculated using the Limma R package.

Among the six SNPs (**Table 2**), three (rs2291236, rs2962375, rs7736442) were located on *BASP1*; rs9273410, *HLA-DQB1;* rs2965298, *R3HDM4;* and rs924150, *TSHZ3*. As shown in **Figure 4**, the gene expression levels of *HLA-DQB1*, *TSHZ3*, and *BASP1* were significantly higher in the case group than the healthy control group (*P* < 0.05), while *R3HDM4* was significantly lower in the case group than in the control group (*P* = 0.006).

**Table 2:** Results of the six SNPs in the GWAS database.

Number	SNPs	mRNA	Chr	Alleles	Cases (N=155)	Controls (N=141)	MAF (Cases)	MAF (Controls)	OR (95%CI)^a^	*P*
1	rs2291236	*BASP1*	chr5:17228908	C>T	88/57/10	103/33/5	0.248	0.152	2.26 (1.34-3.80)	0.002
2	rs2962375	*BASP1*	chr5:17226471	A>T	70/68/17	49/78/14	0.329	0.376	0.52 (0.32-0.84)	0.007
3	rs7736442	*BASP1*	chr5:17241405	C>T	78/60/17	87/47/7	0.303	0.216	2.01 (1.25-3.23)	0.004
4	rs9273410	*HLA-DQB1*	chr6:32627250	C>A	41/78/36	51/68/22	0.484	0.397	1.44 (1.03-2.01)	0.035
5	rs2965298	*R3HDM4*	chr19:90372	G>A	59/75/21	51/62/28	0.377	0.418	0.60 (0.36-0.99)	0.045
6	rs924150	*TSHZ3*	chr19:31829903	A>C	41/82/32	54/64/23	0.471	0.390	1.88 (1.21-2.91)	0.005

a Logistic regression analysis adjusted for age, sex, years of silica dust exposure and smoking status in the additive model (OR; CI).

### Validation Between eQTL-SNPs and Silicosis Based on iMLDR Genotyping Platform

To further validate the relationship between the six eQTL-SNPs and silicosis susceptibility, the iMLDR technique was used for genotyping. The result suggested that the mutant A allele of rs9273410 may increase silicosis susceptibility (additive model: OR= 1.31, 95% CI = 0.99-1.74, *P* = 0.061).

The GWAS screening data were combined with the iMLDR validation data to define the association between rs9273410 and silicosis susceptibility. The two-stage case-control study revealed that the mutant A allele of rs9273410 was associated with increased silicosis susceptibility (additive model: OR= 1.35, 95% CI =1.09-1.68, *P* = 0.006) (**Table 3**).

**Table 3:** Specific information of rs9273410 in the different stages.

Stage	Genotypes	Cases, N (100%)	Controls, N (100%)	Adjusted OR^b^ (95%CI)	*P*
Screening	CC	41 (26.45)	51 (36.17)	1 (ref)	
	CA	78 (50.32)	68 (48.23)	1.54 (0.90-2.64)	0.114
	AA	36 (23.23)	22 (15.60)	2.02 (1.02-4.01)	0.043
	Dominant model			1.67 (1.00-2.77)	0.049
	Recessive model			1.55 (0.85-2.81)	0.150
	Additive model			1.44 (1.03-2.01)	0.035
Validation	CC	46 (24.08)	69 (29.87)	1 (ref)	
	CA	99 (51.84)	120 (51.95)	1.21 (0.76-1.93)	0.412
	AA	46 (24.08)	42 (18.18)	1.74 (0.98-3.07)	0.057
	Dominant model			1.35 (0.87-2.09)	0.186
	Recessive model			1.53 (0.95-2.47)	0.083
	Additive model			1.31 (0.99-1.74)	0.061
Combined	CC	87 (25.14)	120 (32.26)	1 (ref)	
	CA	177 (51.16)	188 (50.54)	1.32 (0.93-1.88)	0.114
	AA	82 (23.70)	64 (17.20)	1.83 (1.19-2.83)	0.006
	Dominant model			1.45 (1.04-2.02)	0.027
	Recessive model			1.53 (1.05-2.22)	0.025
	Additive model			1.35 (1.09-1.68)	0.006

b Logistic regression analysis adjusted for age, sex, years of silica dust exposure and smoking status.

### eQTL Analysis

**Figure 5:**
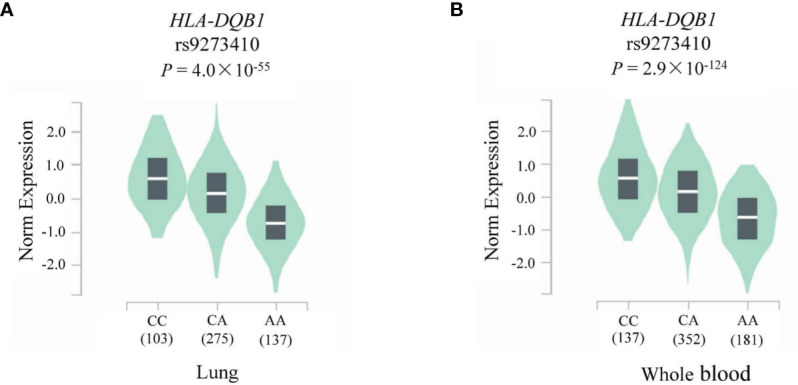
*HLA-DQB1* relative expression levels in the **(A)** lung and **(B)** whole blood according to rs9273410 genotypes.

To assess the functional relevance of rs9273410 on *HLA-DQB1* expression, eQTL analysis was performed using the GTEx database. As illustrated in **Figure 5**, significant associations were observed between *HLA-DQB1* expression and rs9273410 genotypes in both the lung (*P* = 4.0 × 10^-55^) and whole blood tissue samples (*P* = 2.9 × 10^-124^). *HLA-DQB1* expression level of rs9273410 was significantly lower in participants with the CA (or AA) genotype than in those with the CC genotype.

## Discussion

In this multi-stage study, we systematically appraised the association between functional eQTL-SNPs and silicosis susceptibility by integration of RNA-seq data and GWAS mapping. The two-stage case-control study revealed that the mutant A allele of rs9273410 was potentially associated with increased silicosis susceptibility.

Increasing evidence suggests that mRNA is involved in the regulation of various fibrotic diseases. For instance, the interaction between galectin-3 and *ST2* can be used to identify high systemic fibrosis in patients with acute heart failure (22). Moreover, gene-specific hydroxymethylation, which is based on CRISPR/Cas9, can affect gene expression and alleviate renal fibrosis (23). Recently, Junsuk et al. demonstrated that alternative polyadenylation, which is attributed to *NUDT21* reduction, significantly increased the expression of fibrotic mediators and proteins in lung fibroblasts by shortening the 3’-untranslated regions (3’-UTRs) of mRNAs, stabilising their transcripts and eventually activating pathological signalling pathways (24). A study reported that *HDAC8* expression increased in IPF lung tissue, indicating that *HDAC8* expression contributes to pulmonary fibrosis and *HDAC8* inhibition can treat IPF and other fibrotic lung diseases (25). In addition, Liu et al. reported that the upregulation of *NRF2*-mediated LOC344887 contributes to the antifibrotic potential of *SFN* by repressing the expression of *CDH2* and other fibrotic genes (26). These studies indicate that the differential expression of mRNA is associated with the occurrence and development of fibrotic diseases. Therefore, this study aimed to fill the gap between mRNA and silicosis susceptibility.

The identified SNP rs9273410 is located in the 3’-UTR region of *HLA-DQB1.* Recently, an increasing number of studies have reported that *HLA-DQB1* in the MHC region may promote inflammatory response, which is a characteristic of silicosis (27). *HLA-DQB1* has the strongest genetic association with scleroderma because of excessive fibrosis of the internal organs (28). Severin et al. reported that two *HLA-DQB1* alleles (HLA-DQB1*0303 and HLA-DQB1*0609) were significantly associated with the rapid progression of hepatic fibrosis (29). In addition, Louise et al. selected 50,008 unique samples from the UK Biobank and reported six novel genome-wide significant signals of association with the extremes of *FEV1*, including independent signals at two previously reported loci (*NPNT* and *HLA-DQB1/HLA-DQA2*). Their results also revealed that *HLA-DQB1* was associated with chronic obstructive pulmonary disease (30). A recent study indicated that several HLA–tumour–peptide interactions were the major factors modulating lung cancer susceptibility (31). A previous study also revealed a genome-wide significant association between the HLA region and fibrotic idiopathic interstitial pneumonias (27). Our study is the first to report the susceptibility of *HLA-DQB1* for silicosis. Therefore, this study indicates that *HLA-DQB1* may promote lung fibrotic disease development.

The SNP rs9273410 is present in the binding site of miR-888, which is associated with the development of various diseases (32). Huang et al. reported that E-cadherin is directly targeted by miR-888, which exists in MCF-7 side population sphere cells (33). Therefore, the biological rationality indicates that SNP rs9273410 may regulate miR-888 and influence fibrotic disease development through epithelial–mesenchymal transition. In addition, miR-888 was significantly upregulated in lung adenocarcinoma, suggesting that miR-888 plays a role as an oncogene in the progression of lung adenocarcinoma and is a potential therapeutic target for patients with lung adenocarcinoma (32). Further studies investigating the potential mechanisms of rs9273410 that contribute to silicosis *via* miR-888 regulation are warranted.

This study has several merits. First, the combination of GWAS and RNA-seq results helped to identify more precisely the potentially functional SNPs associated with silicosis susceptibility, and the genes that were identified in both the RNA-seq and GWAS had a higher probability of revealing the molecular basis of silicosis development. Second, the functional eQTL-SNP rs9273410 was obtained through the GWAS screening (155 cases and 141 controls) and iMLDR validation (194 cases and 235 controls). This double validation adds value to our results. Third, in the eQTL analysis, we considered both the lung and whole blood tissues, increasing the coverage area of our results compared to previous results when either the lung or whole blood tissues were considered. Finally, in the RNA-seq screening stage, to match the exposure years between the case and control groups, four healthy controls based on 1:1 individual matching on silica dust exposure years and age were selected as controls, thereby enhancing the study’s statistical efficiency. However, some limitations of our study still exist. Firstly, in the GWAS screening and iMLDR validation stages, in order to retain all available cases to evaluate the association between genetic variants and the susceptibility of developing silicosis, we mainly matched the cases and controls by the years of silica dust exposure, while age of the two groups were not comparable. Although we have adjusted for age in the further logistic regression analysis, we cannot exclude the possibility that this factor may affect our results, and further studies with strict matching should be worth attention. Secondly, although this study was designed as a multi-stage study that integrates RNA-seq, GWAS and iMLDR, it was carried out mainly on population-based case-control studies and further functional verification of basic biological experiments should be included to reveal the potential mechanisms.

In conclusion, our study identified an eQTL-SNP rs9273410 located on *HLA-DQB1* that might contribute to the development of silicosis. However, further evaluation of the biological mechanisms of rs9273410 and *HLA-DQB1* is warranted.

## Data Availability Statement

The datasets presented in this study can be found in online repositories. The names of the repository/repositories and accession number(s) can be found below: https://figshare.com/s/4d5a6a115f52f78d3674.

## Ethics Statement

The studies involving human participants were reviewed and approved by the Ethics Committee of Nantong University (Approval No. 2020-002). The patients/participants provided their written informed consent to participate in this study.

## Author Contributions

Conceptualization, YaZ, ZC and YiZ. Data curation, YaZ, YD, and AQ. Formal analysis, RZ, MT and YiZ. Funding acquisition, WW and MC. Investigation, YaZ, YD, AQ, YiZ, and MC. Methodology, YiL, YuL, XZ, and TT. Resources, YiZ, WW, and MC. Software, YaZ and ZC. Supervision, YiL, YuL, XZ, and TT. Visualization, RZ, MT, and MC. Writing—original draft, YaZ, RZ, and YiZ. Writing—review and editing, YaZ and MC. All authors contributed to the article and approved the submitted version.

## Funding

This work was supported by the Natural Science Foundation of Jiangsu Province (BK20191449, SBK2021020088). The scientific research projects of Jiangsu commission of health (Z2019004, H2019020). Jiangsu Association of Science and Technology Youth Science and Technology Talents Enrollment Project in 2019. Postgraduate Research & Practice Innovation Program of Jiangsu Province (KYCX21-3126, KYCX20-2852).

## Conflict of Interest

The authors declare that the research was conducted in the absence of any commercial or financial relationships that could be construed as a potential conflict of interest.

## Publisher’s Note

All claims expressed in this article are solely those of the authors and do not necessarily represent those of their affiliated organizations, or those of the publisher, the editors and the reviewers. Any product that may be evaluated in this article, or claim that may be made by its manufacturer, is not guaranteed or endorsed by the publisher.
